# Supplementary figures and images for: Fixational saccade inhibition and pupil dilation during self-paced limb movement preparation
Source: PLoS One. 2025 Oct 27;20(10):e0335504. doi: 10.1371/journal.pone.0335504 (PMC12558460; doi:10.1371/journal.pone.0335504)

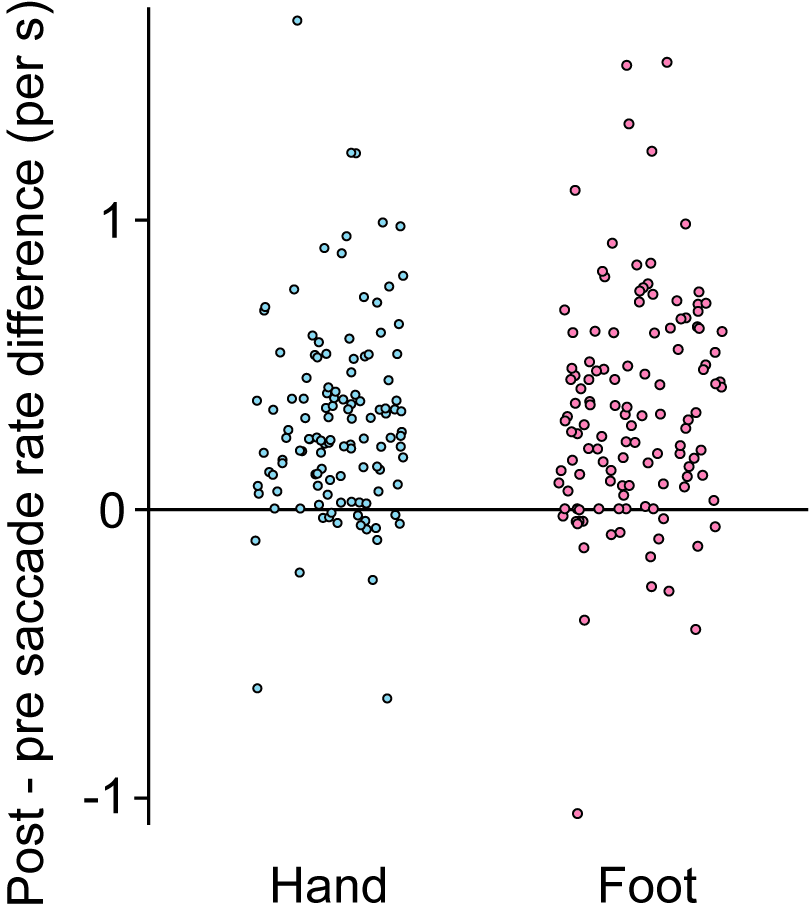

Supplement: S1 Fig — To evaluate the consistency across participants of Fig 3’s pattern of saccade rate inhibition and rebound, we subtracted, for each individual, average saccade rates between two event-related time windows, indicated by the light shaded regions in the plots of Fig 3, with positive numbers indicating higher saccade rates immediately following motor actions than immediately before. These per-observer difference values are summarized here as strip plots, showing that the majority of participants individually show modulation that is qualitatively consistent with the across-observer average of Fig 3. (TIF) [file pone.0335504.s001.tif]

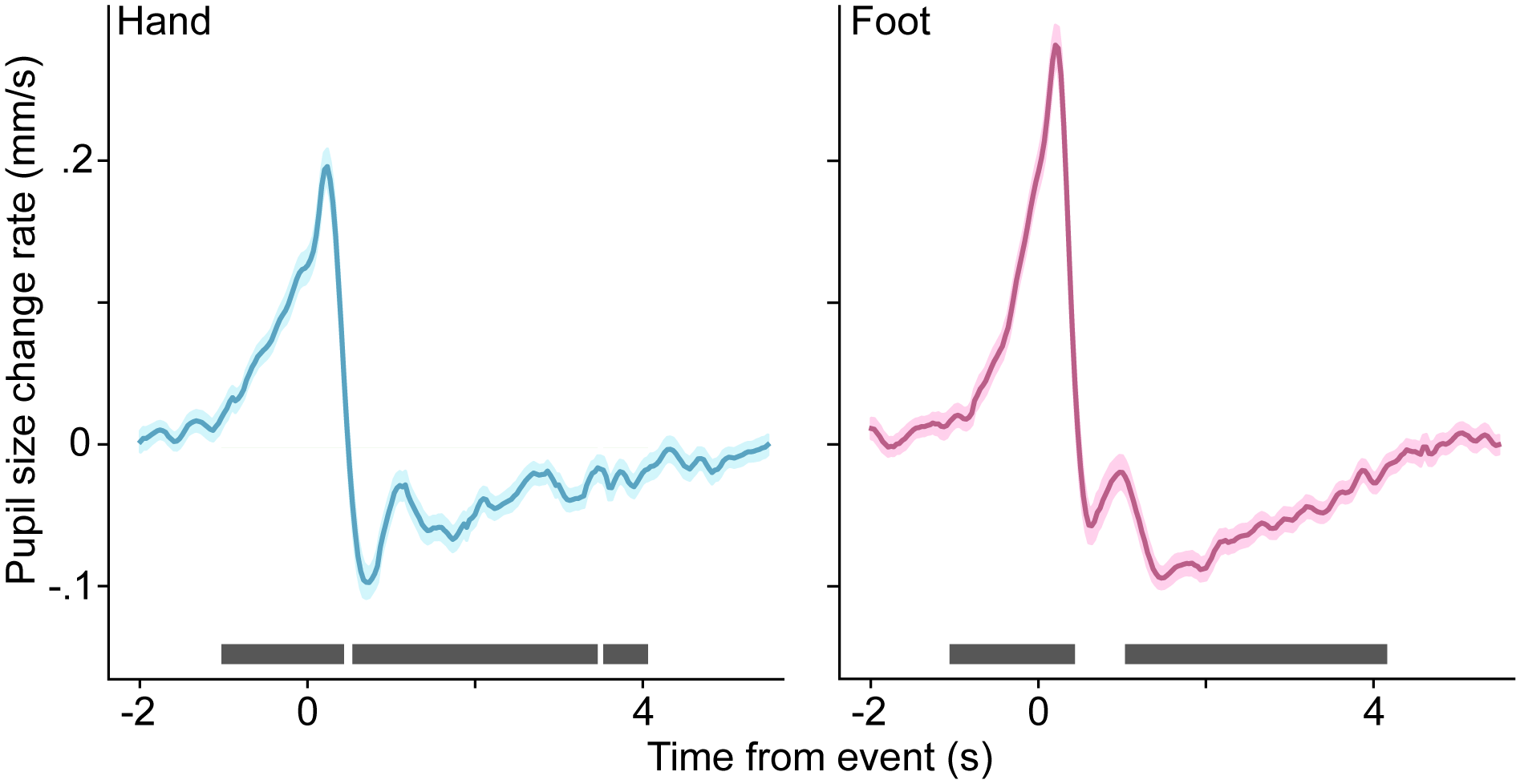

Supplement: S2 Fig — Compared to main text Fig 4, the difference is that the general linear model underlying S2 Fig included oculomor events (saccades and blinks) as nuisance regressors (see Methods). The curves are not identical to those of Fig 4 but do show a very similar pattern, meaning that our results do not importantly depend on this analysis choice. Relatedly, our estimate of the temporal delay between motor-related saccade rate modulation and motor-related pupil size changes (main text, last paragraph before Discussion) remains essentially unaltered when using these curves instead of those of Fig 4 (with these curves, the estimated delay ranges between 238 ms and 313 ms). Dark bars near the x-axis designate periods of significant deviation from 0 mm/s. (TIF) [file pone.0335504.s002.tif]

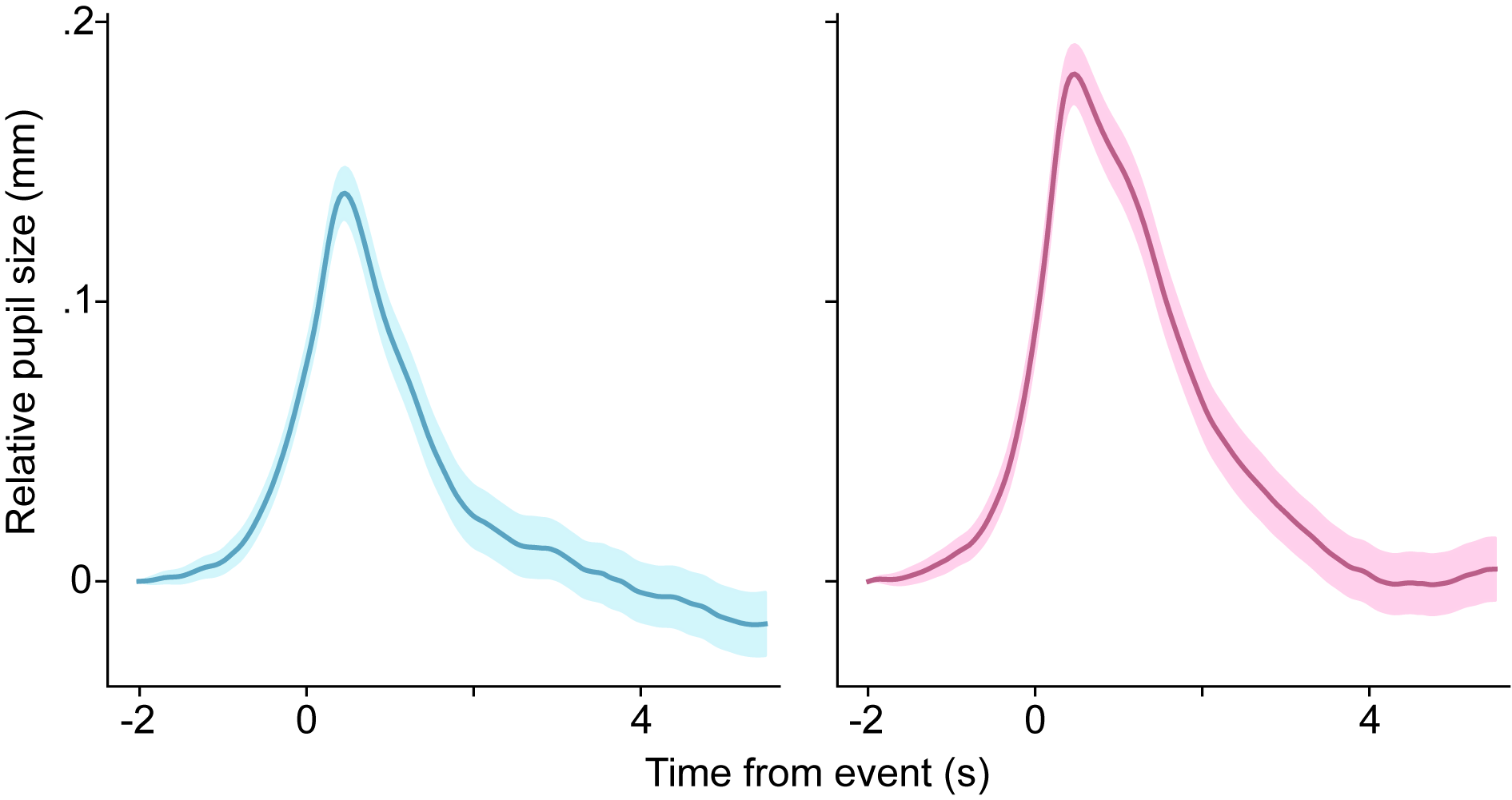

Supplement: S3 Fig — The visualization of pupil data in main text Fig 4 emphasizes the similarity to Fig 3’s saccade rate data. To facilitate comparison to existing work, we replot the data of Fig 4 here, this time showing the cumulative sum (Riemann sum) across the values of Fig 4’s plots, resulting in curves that denote pupil size relative to the start of the time window (as opposed to pupil size change rate). (TIF) [file pone.0335504.s003.tif]
