# Supplementary material for: Fixational saccade inhibition and pupil dilation during self-paced limb movement preparation
Source: PLoS One. 2025 Oct 27;20(10):e0335504. doi: 10.1371/journal.pone.0335504 (PMC12558460; doi:10.1371/journal.pone.0335504)
Supplement: Attachment — Submitted filename: response_to_reviewers.docx [file pone.0335504.s005.docx]

*We thank the Editor and both Reviewers for their input on the manuscript. We have now prepared a revised manuscript as well as a response to the points that were raised. The Reviewer points are copied below, each followed by our response in italics.*

Reviewer #1: In the submitted manuscript, the authors investigate the eye movement dynamics with respect to hand and foot movements. The novelty of their research is that they used spontaneously generated hand and foot movements independent from the environmental cues or task to uncover the coupling between eye movements and limbic movements in isolation. They showed that the pupil dilates, and the saccade rate drops before the onset of an action, and they recover afterwards. They interpret the correlation, timing, and the relative latency of these eye movements with respect to neurophysiological mechanisms associating motor planning with eye movements.
I believe the authors present interesting research, which offers a unique insight on motor coupling isolated from external signals. However, I think their results are occluded by occasional unclarities and overall detailed repetitions in the current manuscript. Conversely, I believe some key details are missing, although I found the paper already long relative to the number of novel results. I recommend that the authors make a major revision to condense their manuscript by reducing repetitiveness, removing excess details, figures, and analyses, and reconsidering the appropriateness of contents of the individual sections. Below, I added point-by-point comments detailing and specifying these suggestions.

*We thank the reviewer for their careful evaluation of the manuscript. We have made changes to accommodate the points raised, including changes that reduce the occurrence of repetitions and improve brevity, as well as changes that should improve clarity. Specific changes are marked below.*

- I believe the introduction and discussion can benefit from introducing Marry Hayhoe’s work on “look-ahead fixations” particularly on hand-eye coordination to introduce the everyday functionality of large saccades with relation to body movements and planning.

*We think this is a good idea. We now address this work in our revised manuscript, lines 56-57.*

- How long/extensive was the training?

*Both tasks were practiced for close to two minutes. We now note this in the revised manuscript (lines 108-109).*

- Please clarify L108-L111

*The lines referenced here, which are lines 115-118 in the revised manuscript, are about the sequence of task runs within blocks. We have now added a clarifying sentence following those lines, at lines 118-121.*

- Please report the sampling rate of eyelink used for binocular tracking. Did they calibrate the eye tracker for individual participants? If so, please describe the calibration process and frequency, i.e. every x trials/blocks.

*Sampling rate was 1000 Hz, and the eye tracker was calibrated at the beginning of each session. We have now added this information (lines 193-198).*

- Where was the fixation point located at the beginning of the experiment? Since the fixation point adaptively repositioned, it would be informative to know the drift from the starting location.

*The fixation point was located at the center of the screen. It did not reposition during the experiment, but we understand how our phrasing may have given rise to that impression. In the revised manuscript we now note the location of the fixation point (line 131) and we have rewritten the section that may have given rise to the impression of adaptive repositioning (lines 156-162).*

- Please describe the "stimulus events" in Methods. I would particularly appreciate details on the factors that could influence the eye movements investigated (e.g., luminance of different stimuli, inter-stimulus-interval, randomization, etc.). Apart from the description pertaining to the pupil light reflex, the reader has very limited information on the visual stimuli. Relatedly, since there were different "events" occurring on the screen, I wonder if participants had similar baseline eye movements for different events.

*We have now added a general description of the stimulus events, which included both visual and auditory events (lines 122-125). Please note that these events happened during different experiment blocks than the ones during which we recorded the data yoked to button presses that are presented in this manuscript: the manuscript's data were recorded while only a fixation point was on the screen, and while participants made self-paced button presses without any stimulus events. This renders it less likely that the stimulus events would impact the button-press related data reported here. Regarding the reviewer's question whether different events would have had similar baseline eye movements: they may not have had similar baseline eye movements. However, please note that this consideration may be rendered less relevant given the above-noted fact that the events did not happen during the time windows during which the here-reported data were recorded, and also given the fact the condition order was identical for all participants. This means that all participants would have had the same task events during blocks immediately preceding the blocks that are of interest for our present purposes. These considerations are now mentioned in the revised manuscript (lines 122-132 and line 113).*

- The experiment seems to impose a very strict control over the participants with visual warnings and dialogs with the experimenters. I wonder if and how these warnings and dialogs influence the spontaneity of subsequent actions. These warnings by themselves can be considered as "events" that alter participants’ oculomotor response. Did the authors check those instances for systematic effects?

*We agree with this assessment about our control of participant behavior. We chose this form of control to strike a balance between motivating participants to engage in spontaneous, unprompted motor actions on the one hand, and on the other hand ending up with uninformative data (for instance, participants tapping a button continuously, which precludes event-related analyses, or pressing a button only once or twice per block, thereby providing very little event-related data). The visual warnings and dialogs likely did influence oculomotor at the time that the warnings and dialogs occurred. We did not check for systematic effects of this impact, because the warnings and dialogs happened between runs, whereas our analysis windows are tied to motor events within runs. Between runs, participants were free to relax, close their eyes, etcetera, so our data are not very suitable for analyzing between-run phenomena or their impact on subsequent within-run data. We have now added more motivation for our choices regarding control of participant behavior to the revised manuscript (lines 138-142).*

- I wonder how the authors made sure that the usb inputs and eye tracker data collected by different devices and software with different latencies are aligned.

*We think this is a very good point. Skeletomotor actions were registered with a likely delay that is worth noting. Our code polled the USB devices every screen refresh, which means every 1/85 s, or every ~12 ms. Peri-event data like those of Figure 2 are plotted relative to the *registered* motor action, and the polling rate means that those types of data may shift rightward slightly (by up to 12 ms) when plotted relative to the actual motor action. We now note the polling rate and its influence on temporal alignment in the revised manuscript (lines 201-202). Please note that none of our conclusions hinge on the fine-grained timing of the key presses. Some of our conclusions do rely on the temporal relation between saccade and pupil events -- gaze and pupil data were recorded by the same device and at a much faster rate of 1000 Hz (see above), so recording inaccuracy in the relative timing of those two types of events is not a concern.*

- I would like to thank the authors for using pupil area change in addition to the loss signal to separate actual blinks from signal loss. If they also checked if blinks were binocular as a criterion, they should mention it.

*Thank you for mentioning this. We did, in fact, also check for binocularity as a criterion. We now mention this in the revised manuscript (line 221).*

- To improve the flow of the paper, I strongly recommend authors provide motivation for their analyses and measurements (e.g. pupil light reflex) in the introduction rather than promising it in the methods for the result section.

*We agree that this improves readability. We have now modified the manuscript accordingly, by adding a final paragraph to the Introduction section (lines 86-93).*

- How did the authors rationalize their selection of time window and box car of saccade rate analysis? What was the typical range of fixational saccade duration?

*The fixational saccades we identified, had a median duration of 19 ms, with an interquartile range of 14 ms. This is now noted in the revised manuscript (lines 417-418). The time window and box car size for the saccade rate analysis were chosen empirically, to meet a trade-off between wishing to resolve the fine-grained time course of saccade rate changes (e.g., the abrupt increase near the moment of the motor action may artefactually appear gradual at larger values of either the time window or boxcar size), and aggregating across sufficient saccades in each bin for measurement noise to remain modest relative to the amplitude of the actual saccade rate modulation (e.g., without the box car, curves like the ones of Figure 2 look quite irregular, and the underlying rate modulation is less obvious). We have now re-run our analyses with time window sizes ranging from 20-50 ms (it is 25 ms in the manuscript), and box car sizes from 150-350 ms (it is 250 ms in the manuscript), and verified that the results remain essentially the same for those ranges.*

- If I understood correctly the authors counted the occurrence of saccades separately for 25 ms bins within the ~5 second time window. I really think that this section would benefit from clearer description. More importantly, I am curious to hear if and how saccades spanning multiple bins are handled. Finally, if the authors believe this is a better approach than simply calculating the saccade rate for a larger time window before/after action and compare it to baseline calculated for the same time window at a different point in the experiments.

*Thank you for pointing out the lack of clarity in this section. We have now re-written it for clarity (lines 237-246). As for saccades spanning multiple bins: we timestamped each saccade with the moment midway between its start point and end point, and saccades were included in a bin if their timestamp fell within that bin. This means that each saccade was assigned to exactly one bin, regardless of whether it spanned multiple. We apologize for not mentioning this in the initial submission; it is now mentioned in the revised manuscript at lines 243-244. (Note that median saccade duration, at 19 ms, was only slightly shorter than bin size, at 25 ms, so that instances of saccades spanning 2 bins were probably common, while instances of saccades spanning 3 or more bins were rare.) In our view, the benefit of analyzing saccade rate in this fashion, compared to an approach based on a larger time window before/after the action, is that it helps show the temporal evolution of saccade rate modulation. In this manuscript, this allows us to identify the similarity between this temporal evolution and that of pupil size modulation, as well as allowing us to estimate the relative delay between those two patterns of modulation. Please note that, in the manuscript, we do transition from the fine-grained analysis to an analysis more similar to what the Reviewer is describing, when we quantify the modulation amplitude per action (Figure 4) or per participant (Figure 2B).*

- L278 Typo "due"
*Fixed.*

- L315 Please explain what is meant by "mirroring".

*Apologies for the lack of clarity. We now explain (lines 353-355): "randomly mirroring or not mirroring each participant's event-related data in the baseline, where 'mirroring' means subtracting the baseline, flipping the sign of the resulting values, and then adding the baseline again."*

- The authors calculate the difference of differences for checking the correlation between pupil size and saccade rate changes before and after an action per effector. As they mentioned, with this type of data there can be various limitations for statistical analysis. Since their data is unlikely to be normally distributed the authors should use a non-parametric test like Spearman's rho or provide normality tests. An alternative would be to calculate the slope instead of correlation, which would give direction and strength.

*This is a good point. We have now removed the correlation analysis altogether, motivated by the Reviewer's next point. This means we only include the multilinear regression analysis. Contrary to Pearson correlation, multilinear regression does not assume normality of the data.*

- I am genuinely puzzled as to why authors did the correlational analyses if they have thought of potential confounding factors. For clarity and brevity, I strongly recommend them to use one statistical analysis per research question. For the multilinear regression selection of pupil size or saccade rate as the response seems to be arbitrary. If the authors want to account for the tonic pupil size in the saccade-pupil relationship, they could do a partial correlation to remove the variance common to tonic pupil size. However, I believe they should keep one of the analyses.

*We agree that the correlation analysis is redundant given the multilinear regression analysis that includes potential confounding factors. We have now removed the correlation analysis.*

- L386 please give saccade/sec for comparability with the wider literature.

*We have now added this information (line 415). The rate is 0.89 saccades per second.*

- I believe the consideration of individual differences in the results section should be mentioned in the discussion. The related results in Figure 2B could be moved to the supplementary materials for brevity, as the authors neither conduct further analysis based on it nor use it for the interpretation of the results.

*Agreed. We have now moved Figure 2B to the supplementary materials, and only make brief reference to its main point in the main text (lines 430-435).*

- In my opinion, the comparison of results with other studies (L417 onwards) are better kept for discussion to preserve the flow of the study findings.

*We have now removed this comparison, except for a brief reference to the literature's pupil findings to set up the next analysis (lines 434-441).*

- Again, I believe re-plotting the data for the sake of comparability in the result section distracts the reader and it would be better appreciated in supplementary.

*We see the importance of brevity and flow, and we have now moved the re-plotted curves (i.e., the ones with the cumulative sum of the derivative curves) to the supplement.*

Reviewer #2: The authors describe results of a study investigating microsaccade rates and pupillary changes surrounding self-initiated limb movements (arm, foot). They find a signficiant modulation of microsaccade rate and pupil size change, consistent with earlier works. Interestingly, both microsaccade rate and pupil dilation were linked: The more pupil dilation, the stronger the saccadic suppression. While the differing intervals for the motor responses are a bit odd at first, this nicely excludes concurrent effects of external cues on both pupil size and microsaccade rates.I found the manuscript interesting to read. I have a number of points that may help streamline manuscript and visualization though as the manuscript, in present shape, is less straight-fowrard than necessary. Most importantly, I believe that a third (better?) possibility should be considered as explanation to the authors’ findings. This may be at odds with a mere motor inference interpretation.

In order of points in the manuscript:
Can the authors add a clear paradigm figure showing screens etc? Right now the reader has to infer a lot from the text with room for misinterpretations. I was not particularly concerned of a visual event causing the effects (as the authors also expressed in their control analysis), but a visualization of the screen would be helpful to that end.

*We have now added a figure (the new Fig 1) that shows the screen during the task blocks dedicated to spontaneous motor action, and during the task blocks dedicated to measuring the pupil light reflex.*

P7., ; 139 following: I must admit I found the central gaze check rather odd. 500ms are long, also for blinks. It is unclear to me why the gaze check is sensible to conduct only in the horizontal plane then, as regular blinks should not take substantially longer. Even if so, one may question the use of such data as the intervals interpolated become excessive? Did the authors calibrate the pupil to millimeters alone or also apply the PFE calibration described in the paper by Hayes and Petrov? If they did not do the latter, it might be sensible to add a control analyses (perhaps in a supplement) that uses gaze position as a covariate.

*We agree that our central gaze check was more liberal than it could have been. As the gaze check influenced the course of the experiment, we cannot change it retroactively at this point. We agree, however, that blinks tend to take shorter than 500 ms, which alleviates the reviewer's concern about excessively long intervals of interpolated blink data. We have now analyzed the distribution of blink durations in our data, and confirm that our blinks were generally shorter than 500 ms: median: 164 ms, interquartile range: 70 ms. We did not apply the PFE calibration described in the Hayes and Petrov paper, which deals with foreshortening effects on recorded pupil size using video-based eye trackers. We have now performed control analyses to address the concern that our reported movement-related pupil signal, in reality, reflects foreshortening related to systematic changes in gaze position during the peri-movement time window. Specifically, we performed event-related analyses of gaze position relative to the moments of motor action, analogous to our main text's analyzes of saccade rates and pupil sizes relative to those moments. If our observed event-related pupil size changes are artifactual and due to systematic event-related differences in gaze position, then we should find that gaze position surrounding a motor action follows a time course that is similar to pupil size. Our new analyses of gaze position do not confirm this. This plot summarizes the result of one such analysis:*

*The plot shows average absolute gaze distance from fixation as a function of time relative to hand (green) and foot (red) movements. (Note that the y-axis unit here is pixels, and that 27 pixels corresponds to about one degree of visual angle). This absolute distance clearly shows systematic variation during the time window. The time course, perhaps unsurprisingly, is similar to that of fixational saccade rate shown in the main text: average distance from fixation is smallest immediately prior to the motor action, when fixational saccade rate is lowest, and average distance is largest right after the motor action, when fixational saccade rate is highest. While this pattern is consistent, then, with the fixational saccade rate pattern observed in this study, it does not explain the pupil size pattern observed in this study. First, the overall vertical range this plot is much too small to explain the observed variation in pupil size: in terms of standard errors, the overall range of the plot here is around two standard errors, while the peri-event range in pupil size (Fig S3) covers almost ten times as many standard errors. In terms of real-world units, the range in gaze positions plotted here is about 7 minutes of arc (3 pixels between post-event peak and pre-event trough), and we find a maximum pupil dilation of about 5% (0.1-0.2 mm). The Hayes and Petrov study, in contrast, found apparent dilations of that magnitude only for gaze displacements of several degrees (over 10 times more than the 7 minutes of arc range seen in this plot). Finally, the shape of the peri-event gaze curve does not match that of the peri-event pupil size curve (Fig S3). While pupil size starts rising about 1-2 s before the event and stays above baseline until about 3 s after the event, gaze distance from fixation goes down to its minimum, up to its peak, and back down to baseline within that same period. All these observations argue against the notion that the change in pupil size that we observe surrounding limb movements, is an artefactual result of movement-related changes in gaze position. (Please note that we also performed this analysis with other measure of gaze position than absolute distance from fixation, namely both absolute and signed horizontal distance, and both absolute and signed vertical distance. All those analyses gave results similar to the one shown here.)*

P11., l222: This may benefit from some general information on response latencies of pupil size. Bergamin & Kardon, 2003 may provide such numbers. In our own data, we usually observe times around 300-350ms. Similarly, response latencies for externally triggered saccades may be of interest, so that both timings can be compared.

*We have now added a sentence, with citations, comparing the temporal characteristics of the pupil response and saccade rate response (line 274-276): "For example: the constriction elicited by a simple light flash may span from about 300 ms to 1500 ms following the flash (Barbur et al., 1998), whereas microsaccade rate modulation is usually over within 500 ms following such stimuli (Rolfs, 2009).".*

P.13 From a reader’s perspective, the section on the pupil light reflex comes totally out of the blue. I personally don’t think it flows well to cross reference to a later section for motivation. Please motivate clearly how this is needed. If needed, results should be accompanied by a figure ideally.

*We have now changed the text so that the analysis of the pupil light reflex is first motivated and announced in the Introduction section (lines 90-93), so that the analysis does not come as a surprise in the Methods section. It also means that the Methods section (lines 177-191) no longer cross references a later section for motivation, but rather refers back to the Introduction section. We opted not to include a figure for this analysis, because this particular analysis consists of only a comparison between the response delay for the pupil light reflex and the saccade-pupil delay for motor actions.*

P. 18. The first two sentences of the results are repeating parts of the methods, some oddly specific, some very general, I would remove these. Generally, there are many cross references (to methods in the results, to results in the methods) that should be avoided in my opinion.

*We agree that those first two sentences were only repetition, and we have now removed them. We also made various other changes throughout the manuscript to improve flow and readability, including removing of some other repetitions between Introduction and Results, and moving a few figure panels to the Supporting Information.*

Motor responses are indeed very reliably found to affect pupil size, Richer & Beatty is a good reference for that. However, similar effects have been described also substantially earlier by Bumke among others (1911, cf Loewenfeld 1999, Strauch 2024, TINS) which might be worth noting.

*Thank you for pointing us to that reference. We were not aware of it. We now cite it in the revised manuscript (line 82).*

The authors sometimes call microsaccade rate ‘saccade rate’ If I’m not mistaken. I would advise to use ‘microsaccade rate’ consistently if that’s what they calculated (explicitly discarding saccades I believe), see Figure 2, caption.

*We have now changed the text to consistently use the term 'fixational saccade rate'.*

P20. L410 to 415 can be cut in my opinion. The authors do not need to describe how a boxplot works. Ideally, the authors should convert this figure to a stripplot that displays all data points.

*A fair point. We have now removed that description of the interpretation of the box plot, and also replaced the box plot with a strip plot. (And moved the plot to the Supporting Information based on a recommendation by the other Reviewer: the strip plot can now be found in figure S1).*

I wondered about the statistics reported on p23. While the effects are clear, it seems that they underuse the data. Wouldn’t it be better to perform a linear mixed effects model the predicts the change in saccade rate (or number of saccades per interval using an ordinal linear mixed effects model) from pupil size and the other predictors? Or vice versa pupil size from microsaccade rates and other predictors? That way, the authors could also simply take deviations in gaze position, blinks etc into account per trial.

*The statistics referenced in this comment pertain to the correlation analysis that was in the original submission. We have now removed that correlation analysis in response to a comment by the other Reviewer, who correctly pointed out that the multiple linear regression (originally reported in Fig 4B; now in Fig 5) made the same point as the correlation analysis, but better.*

For Figure 4, I think it would make the manuscript a lot nicer to plot faint regression lines per participant (i.e., within a participants’ trials) together with an overall regression line. Of course there will be substantial variation, but this may help assessing the robustness and consistency of the effects, akin to the proposed stripplot.

*The removal of the correlation plot, mentioned at the previous point, is also relevant here. In response to this remark, we did explore the option of plotting the standardized regression coefficients of the multiple linear regression (i.e., Fig 5) as box plots, strip plots, or as bar charts overlaid with the individual data points. All options would have provided more information about the consistency of the effects than a bar chart with standard errors does, but still we decided against all these options in favor of the bar chart with standard errors, because the variation across participants is so large that visually showing this variation forces the y-axis to be scaled down to a point where it is no longer visually clear that the across-participant average does, in fact, differ significantly from zero (for the pupil size predictor variable). For this reason, we decided to stick with the bar chart with errors, but we now provide summary statistics about across-participant spread in the manuscript text (lines 502-506): "Note that, in spite of this highly significant evidence for an association when aggregating across participants, the regression coefficient for event-related pupil size modulation did show substantial variability among participants (for hand and foot, respectively, the median coefficients were 0.077 and 0.079, the inter-quartile ranges were 0.28 and 0.26, and the proportions of participants with a value greater than 0 were 0.66 and 0.72)."*

The authors, in the discussion, speculate about candidate regions in the brain that may underlie the observed link. Personally, I think that this could be a bit more concise, given that the present study allows only limited inference on brain regions (causally) involved beyond primary motor cortex, LC, SC perhaps.

Lastly and perhaps most importantly, I would like to present a third explanation to the authors’ findings. Perhaps it is not that motor actions specifically that interfere with (microsaccadic) eye movements. There is a broad literature demonstrating a perhaps surprising more general link between pupil dilation and (micro)saccade rates. E.g., as the authors will know, pupils dilate with any increase in effort, be it mental or physical (e.g. Bumke 1911, Hess & Polt, 1964, Mathot 2013, Strauch et al., 2022, etc.). Similarly, microsaccade rates drop under increased mental effort (<https://doi.org/10.1111/ejn.12395>), by now an almost as well replicated finding as for pupil dilations. I therefore consider a motor-specific explanation as quite unlikely – why would this link then also extend to mental effort (see also for another example <https://doi.org/10.1167/jov.25.4.16>)? These results may be (more) parsimoniously unified however, if one looks at them from a processing bottleneck or dual-task perspective. Assuming that any (including eye) movement is effortful due to its computation and execution costs, results are to be expected. For instance, we showed that saccades, just as larger scale movements, are linked to movement-specific pupil dilations indicating that they require differing degrees of effort (<https://doi.org/10.7554/eLife.97760.3> , <https://doi.org/10.1177/09567976231179378>). These effort-signatures in turn are quite indicative of behavior, i.e., humans make cheaper eye movements when given the choice (<https://doi.org/10.7554/eLife.97760.3> , see also <https://doi.org/10.1101/2022.06.03.494508>). If, in line with the previously mentioned papers, all movements, arm, leg, eye are considered as effortful, the here demonstrated results make perfect sense: when one movement (or mental operation) requires more computational resources, likely inferable via LC activity and thus pupil size change, then they will interfere with each other (as the present manuscript shows). I personally consider this a more likely explanation to the authors’ results than the present interpretations (although they go a little bit in this direction too) and believe that the paper might make an additional valuable contribution to that literature (but I’m biased here, of course).

*Thank you for pointing us to this literature. While our original submission had argued for a connection that was more general than some proposals in the literature by going beyond just oculomotor action and encompassing skeletomotor action as well, we agree that an even more general connection (in terms of any kind of effort) is possible and perhaps more parsimonious. We have now updated our Discussion section, both to slim down the interpretations we had previously proposed (in response to the Reviewer's point about conciseness), and to add the broader interpretation proposed by the Reviewer as a third option (lines 584-592 and 604-619). We also updated the Abstract to reflect this change.*

I could not open the data repository upon clicking on the doi, so could not check data or scripts.

*Datadryad informed us that some fields needed to be updated before they could publish our materials. We are working on that.*
